# Co-occurrence of hypertension and type 2 diabetes: prevalence and associated factors among Haramaya University employees in Eastern Ethiopia

**DOI:** 10.3389/fpubh.2023.1038694

**Published:** 2023-07-11

**Authors:** Aboma Motuma, Tesfaye Gobena, Kedir Teji Roba, Yemane Berhane, Alemayehu Worku, Lemma Demissie Regassa, Abebe Tolera

**Affiliations:** ^1^School of Nursing and Midwifery, College of Health and Medical Sciences, Haramaya University, Harar, Ethiopia; ^2^Department of Environmental Health Science, College of Health and Medical Sciences, Haramaya University, Harar, Ethiopia; ^3^Department of Epidemiology and Biostatics, Addis Continental Institute of Public Health, Addis Ababa, Ethiopia; ^4^Department of Epidemiology and Biostatistics, School of Public Health, Addis Ababa University, Addis Ababa, Ethiopia; ^5^School of Public Health, College of Health and Medical Sciences, Haramaya University, Harar, Ethiopia

**Keywords:** co-occurrence, hypertension, diabetes mellitus, university employees, Ethiopia

## Abstract

**Background:**

Both hypertension (HTN) and diabetes are public health concerns in low- and middle-income countries, particularly in sub-Saharan African countries. The co-occurrence of HTN and diabetes is associated with an increased risk of mortality, morbidity, and reduced productivity in the working force. In Ethiopia, there is limited evidence on the co-occurrence of HTN and type 2 diabetes (T2DM). Therefore, this study was conducted to assess the co-occurrence of HTN and T2DM and their associated factors among Haramaya University employees in Eastern Ethiopia.

**Methods:**

A cross-sectional survey was conducted among 1,200 employees at Haramaya University using a simple random sampling technique from December 2018 to February 2019. Demographic and behavioral factors were collected on a semi-structured questionnaire, followed by measurement of anthropometry and blood pressure. Blood glucose and lipid profile measurements were performed by collecting 6 ml of venous blood samples after 8 h of overnight fasting. Data were entered into EpiData 3.1 version and analyzed using Stata 16 software. Bivariable and multivariable logistic regressions were applied to observe the association between independent variables with co-occurrence of HPN and T2DM using odds ratio, 95% confidence interval (CI), and *p*-values of ≤ 0.05 were considered statistically significant.

**Results:**

The prevalence of HTN and T2DM was 27.3 and 7.4%, respectively. The co-occurrence of HTN and T2DM was 3.8%. The study found that being older (AOR = 3.97; 95 % CI: 1.80–8.74), khat chewing (AOR = 2.76; 95 % CI: 1.23–6.18), body mass index ≥ 25 kg/m^2^ (AOR = 5.11; 95 % CI: 2.06–12.66), and sedentary behavior ≥8 h per day (AOR = 6.44; 95 % CI: 2.89–14.34) were statistically associated with co-occurrence of HTN and T2DM. On the other hand, consuming fruits and vegetables (AOR = 0.10; 95 % CI: 0.04–0.22) and a higher level of education (AOR = 0.39; 95% CI: 0.17–0.89) were negatively statistically associated with the co-occurrence of HTN and T2DM.

**Conclusion:**

The co-occurrence of HTN and T2DM was prevalent among the study participants. This may create a substantial load on the healthcare system as an end result of increased demand for healthcare services. Therefore, rigorous efforts are needed to develop strategies for screening employees to tackle the alarming increase in HTN and T2DM in university employees.

## Introduction

Non-communicable diseases (NCDs) are the primary causes of morbidity and mortality globally. It causes 41 million death each year, equivalent to 71% of all deaths globally (1, 2). Almost 75% of all disease deaths and 82% of the 16 million people who died prematurely or before reaching 70 years of age occur in low- and middle-income countries (3) including 39.3% in Ethiopia (4).

Hypertension (HTN) and diabetes (DM) are global public health problems (5). They have been confirmed as two of the major risk factors for cardiocerebrovascular diseases as leading causes of death and disability among adults (6). It has been found that individuals with both HTN and DM have a greater risk of cardiocerebrovascular disease than those with only one condition (7). Hypertension (HTN) and DM are also the challenges of the healthcare system in low- and middle-income countries (8), due to changes in their diet habits and lifestyle and increased sedentary behavior (9, 10). Hypertension (HTN) and DM share common comorbidities and risk factors (11). Complications related to HTN and DM are reduced quality of life and productivity (12), such as the increased risk of stroke (13), cardiovascular diseases, end-stage renal disease (14, 15), retinopathy, depression, impaired health-related quality of life, and increased healthcare costs (16–18).

The coexistence of HTN and type 2 diabetes (T2DM) has been documented in previous studies (19, 20). Epidemiological studies have documented that DM predisposes to HTN (16, 17). For instance, a study in the USA showed that up to 75% of adults with diabetes also have hypertension (21), and in China, ~15 million people have both HTN and DM (22).

In Ethiopia, about 1.7 million cases and 23,157 deaths were related to DM in 2019 (23). Moreover, evidence shows that the pooled prevalence of HTN was 21.8%, which was the highest prevalence reported in Addis Ababa at 25.4% and the lowest in the Tigray region at 15.4% (24). In Ethiopia, reports indicate that among university employees and civil servants in Addis Ababa, the prevalence of HTN was 13.9 and 27.3%, respectively (25, 26). Similarly, studies showed that DM among university employees and governmental civil servants at Guji Zone was 4.7 and 3.9%, respectively (25, 26). Moreover, studies showed that co-occurrence of HTN and T2DM accounted for ~40–75% of patients (27).

Although the co-occurrence of HTN and T2DM is believed to be prevalent among university employees in Ethiopia, there is limited evidence. Therefore, this study aimed to assess the co-occurrence of HTN and T2DM among university employees in Eastern Ethiopia.

## Materials and methods

### Study area

This study was conducted among Haramaya University employees from December 2018 to February 2019. Haramaya University is located ~510 kilometers east of Addis Ababa, Ethiopia. The university has ~7,176 employees, 28.1% women and 71.9% men. In terms of job mix, 21.1% of the staff are academic and the other 78.9% are grouped under technical and administrative staff.

### Study design and period

An institutional-based cross-sectional study was conducted among 1,200 university employees who were randomly selected. The study participants in the age range of 18 to 65 years and who were working for at least 6 months were included. Self-declared physical disability and pregnant women were excluded from the study. A single population proportion formula was used to calculate the sample size using OpenEpi 3.1. based on a previous study in China (22); double burdens of hypertension and T2DM were estimated as 3.8% with a 95% confidence level (the critical value Zα/2 = 1.96), a 2% margin of error, and a 10% non-response rate. Finally, a total of 1,200 study participants were recruited. The sample was drawn using simple random sampling techniques based on the proportion to the size of their respective departments using the payroll roster as the sampling frame.

### Data collection and tools (questionnaire)

Data were collected by trained nurses and laboratory technicians using a structured questionnaire adapted from the WHO STEPS, through face-to-face interviews, physical measurements, and biochemical tests. A pre-test was conducted on 5% of the final sample size at another nearby public university. Data collectors were trained on how to conduct interviews, anthropometric measurements, and field data collection procedures before directing them to gather data needed from data providers. Then, study participants were appointed on the next day morning in fasting condition to collect blood samples at the university clinics by trained medical laboratory technologists. The field research supervisors closely supervised the data collection process and checked the completeness and accuracy of the filled data on a daily basis. A field guide and data collection manual were used as a reference during the training. Standard operating procedures (SOP) were followed starting from the sample collection up to reporting of the result.

### Variables and measurements

The dependent variable is the co-occurrence of hypertension and T2DM. Blood pressure was measured using a digital measuring device (Microlife BP A50, Microlife AG, Switzerland) with participants sitting after resting for at least 10 min. Then, three times blood pressure measurements were taken in an interval of at least 5 min between consecutive measurements. In doing so, the mean systolic and diastolic BP from the second and third measurements was analyzed and diagnosed as hypertension when the mean systolic and diastolic blood pressure was greater or equal to 140/90 mm Hg (28). To determine blood glucose level and lipid profile, 6 ml of venous blood samples was collected from the participant's antecubital arm in a seating position after 8 h of overnight fasting. The collected samples were then directed into the sterile vacuum tube (Gel Clot Activator) and placed on the rack for 10–20 min for clotting formation.

The specimen tubes were then centrifuged at 3,000 revolutions per minute to extract the serum from the whole blood; fasting serum triglyceride and blood sugar were analyzed using the standard enzymatic colorimetric method (HUMAN Gesellschaft fur Biochemica und Diagnostica mbH Max-Planck-Ring, Germany), while HDL-c was analyzed using direct homogenous standard enzymatic assay colorimetric test (HUMAN Gesellschaft fur Biochemica und Diagnostica mbH Max-Planck-Ring, Germany) (29). Lipid profile and fasting blood glucose concentrations were reported in mg/dL (30). The prevalence of T2DM was defined as fasting blood glucose of ≥126 mg/dl or a self-reported diagnosis of diabetes (31).

Smoking status was categorized as never-smoking, current smoking (at least one cigarette per week), and former smoking (quit smoking more than 12 months) (32, 33). Current drinkers were those who drank once or more in the prior month of data collection. The habit of using Khat (Catha edulis) was grouped into never, occasional, and habitual (frequent). The total physical activity score was computed as the sum of all metabolic equivalent (MET)-minutes per week for vigorous-intensity physical activity, moderate-intensity physical activity, and walking. The sum of MET-minutes per week was categorized as high (3,000 MET-minutes or above), moderate (between 2,999 and 600 MET-minutes), and low (< 600 MET-minutes).

Anthropometric data (weight, height, waist, and hip circumference) were collected according to the WHO STEPS manual (28). A BMI of ≥25.0 was defined as overweight/obese according to the WHO STEPS (31). Waist circumference was measured midway between the costal margin and the iliac crest with a tape line (31).

### Statistical analysis

Data were double-entered into EpiData version 3.1 and cleaned, coded, and then transferred to STATA version 16 for analysis. The data were checked for missing values and outliers. The magnitude of hypertension, T2DM, and co-occurrence were summarized using proportion and frequency. A binary logistic regression model was used to determine factors associated with the co-occurrence of hypertension and T2DM. Information criteria (AIC/BIC) were used to select the final optimal model. The multicollinearity was checked using a scatter matrix and VIF. Interaction between variables was also checked, and the effect of omitted variable effect was tested using “ovtest.” Model fitness was checked with Hosmer–Lemeshew test, overall model statistics were checked with “fitstat” command, and the *p*-value of the Hosmer–Lemeshew was found to be 0.25. The magnitude of variables with a *p*-value of < 0.25 in bivariate analysis results obtained were taken for the multivariable analysis. Associations between outcome variables and covariates were reported with an adjusted odds ratio with a 95% confidence interval, and finally, statistical significance was considered at a *p*-value of < 0.05.

## Results

### Socio-demographic characteristics of the study participants

A total of 1,200 employees were enrolled, with 1,164 providing data, resulting in a 97% response rate. The majority of study participants were men, 598 (51.4%) resulting in a sex ratio of 1:1.06. The mean age of the study participants was 35 (±9.4 SD) years, with a range of 20–60 years. In the majority of the study participants, 755 (64.9%) were non-office workers and 739 (63.5%) attended college and above education level. The mean reported per capita annual income of participants was 1,05,059.1 (±49,960.38 SD) (Table 1). Nearly 37 and 33% of study participants had high total cholesterol and triglyceride level.

**Table 1:** Socio-demographic characteristics of civil servants of Haramaya University in Eastern Ethiopia, 2019 (*n* = 1,164).

**Socio-demographic variables**	**Category**	**Frequency (*n*)**	**Percent (%)**
Sex	Male	598	51.4
	Female	566	48.6
Age in years	< 45	941	80.8
	≥45	223	19.2
Occupation	Non-office worker	755	64.9
	Office worker	409	35.1
Level of education	Primary school (grade 1–8)	193	16.6
	Secondary school (grade 9–12)	232	19.9
	College and above (grade 12+)	739	63.5
Monthly salary income in ETB	< 2,000	367	31.5
	2,000–4,000	328	14.4
	4,001–6,000	168	28.2
	>6,000	301	25.9
Ethnicity	Oromo	509	43.7
	Amhara	549	47.2
	Others^a^	106	9.1
Marital status	Never married	427	36.7
	Married	667	57.3
	Divorced/ widowed	70	6.0
Religion	Orthodox	722	62.0
	Muslim	219	18.8
	Protestant	197	16.9
	Others^b^	26	2.3

^a^Gurage, Tigraway, Harari, and Wolaita.

^b^Catholic, Traditional; ETB, Ethiopia Birr.

Approximately half of the study participants, 571 (49.1%), had low physical activity and, 561 (48.25), were alcohol consumers. Moreover, 396 (34.5) were khat chewers, and 413 (35.5%) were overweight/obese. In addition, 131 (11.3) of the study participants were ever smokers. Table 2 shows details of the anthropometric, biochemical, and lifestyle characteristics of the subjects.

**Table 2:** Biochemical, anthropometric, and lifestyle characteristics of the study participants of Haramaya University employees, Eastern Ethiopia, 2019 (1,164).

**Variable**	**Category**	**Frequency (*n*)**	**Percent (%)**
LDL	Normal (< 130 mg/dl)	903	77.6
	High (≥ 130 mg/dl	261	22.4
Total cholesterol	Normal (< 200 mg/dl)	736	63.2
	High (≥ 200 mg/dl)	428	36.8
HDL	Desirable (≥ 50 mg/dl)	912	78.4
	Low (< 50 mg/dl)	252	21.6
Triglycerides	Normal (< 150 mg/dl)	785	67.4
	High (≥ 150 mg/dl)	379	32.6
Waist circumference	Normal	619	53.2
	High	545	46.81
Body Mass Index	< 25 kg/m^2^	751	64.5
	≥ 25 kg/m^2^	413	35.5
Khat chewing	Yes	398	34.0
	No	768	66.0
Physical activity level	Low ( ≤ 600 MET min/week)	226	19.4
	Moderate (600–2,999 MET min/week)	367	31.5
	High (≥3,000 MET min/week)	571	49.1
Smoking status	Yes	151	11.3
	No	1,033	88.7
Alcohol consumption	Yes	561	48.2
	No	603	51.8
Serving fruits and vegetables	0 servings per day	56	4.8
	At least one-time servings per day	1,108	95.2
Sedentary behavior	< 8 h per day	928	79.7
	≥8 h per day	236	20.3

### The prevalence of HTN and T2DM

The mean systolic and diastolic blood pressure were 124.4 (±16.6SD) mmHg and 79.5 (±10.5SD) mmHg, respectively. The overall prevalence of HTN was 290 (27.3 %); of which, 128 (28.3 %) were women and 162 (27.1 %) were men. Of these, 246 (21.1 %) were previously unknown that they had hypertension, and only 44 (3.8%) were known hypertensive individuals. The mean and highest values of fasting blood glucose were 87.69 (±29.62SD) mg/dL and 341 g/dL, respectively. This study shows that the overall prevalence of T2DM (≥ 126 mg/dL) after overnight fasting was 86 (7.4 %); of which, 40 (7.1 %) were women and 46 (7.7 %) were men. From these, 2.3% were known T2DM individuals and 5.1% were individuals with undiagnosed T2DM.

### Co-occurrence of HTN and T2DM and associated factors

**Figure 1:**
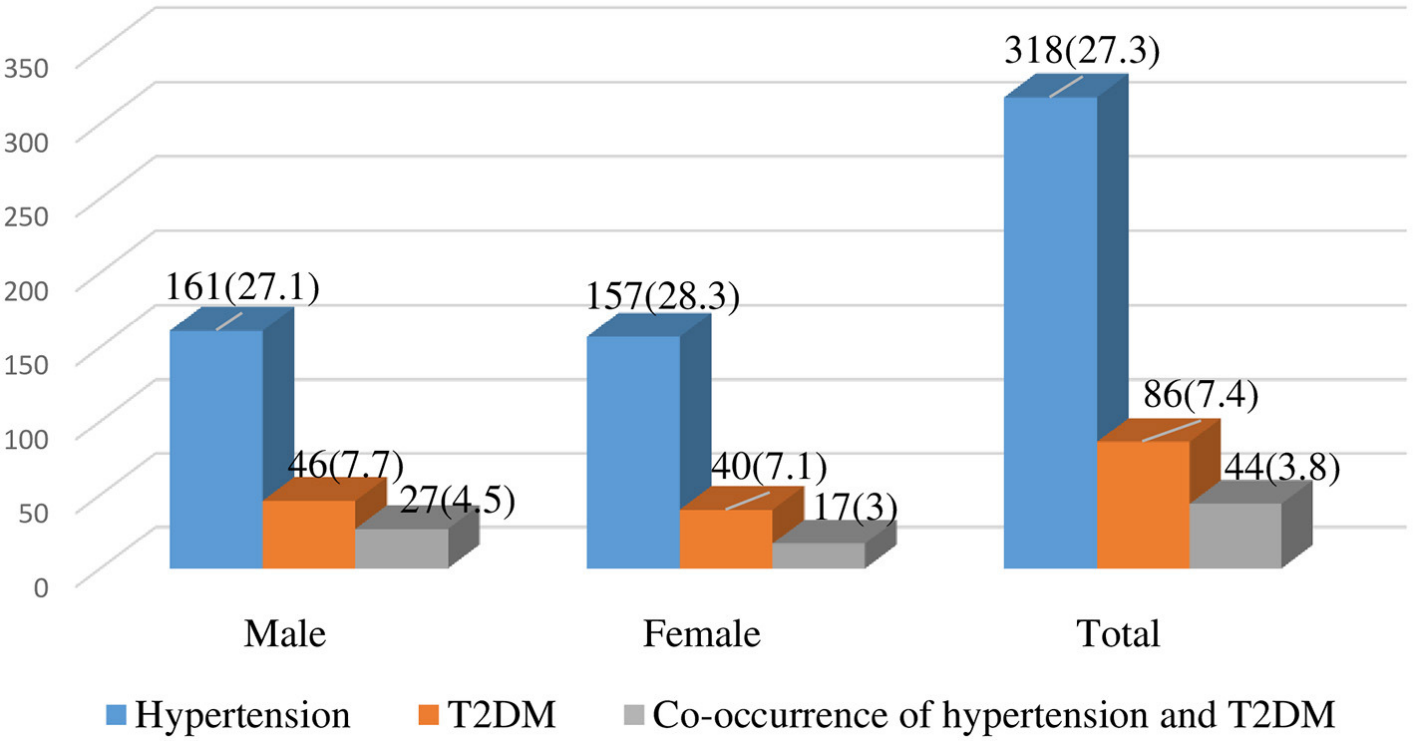
The prevalence of HTN, T2DM, and co-occurrence of HTN and T2DM among Haramaya University employees, Eastern Ethiopia, 2019.

Of the total study participants, 44 (3.8%) had both HTN and T2DM. Of these, 4.5% who had co-occurrence of HTN and T2DM were men (Figure 1).

In multivariable logistic regression analysis, age, intake of fruits and vegetables, khat chewing, educational status, BMI, and sedentary behavior were significantly associated with the co-occurrence of HTN and T2DM.

Participants aged 45 years and above were almost four times more likely to have the co-occurrence of HTN and T2DM compared to participants aged younger than 45 years. Employees who attended college and above were 61% less likely to have the co-occurrence of HTN and T2DM than those who attended less than or equal to secondary school. Khat chewers were nearly three times more likely to have the co-occurrence of HTN and T2DM as compared to non-khat chewers. Moreover, the odds of co-occurrence of HTN and T2DM were five times higher among those whose BMI was >25 kg/m^2^ compared with those whose BMI was < 25 kg/m^2^. Similarly, the odds of co-occurrence of HTN and T2DM among participants having a sedentary lifestyle was approximately 6 times higher than those who do not live a sedentary life. On the other hand, study participants who had servings of fruits and vegetables at least once per day were 90% less likely to have co-occurrence of HTN and T2DM as compared to those who did not consume servings of fruits and vegetables (Table 3).

**Table 3:** Factors associated with co-occurrence of hypertension and type 2 diabetes among Haramaya University employees, Eastern Ethiopia, 2019 (*n* = 1,164).

**Characteristics**	**Variable**	**HTN&T2DM co-occurrence**		**COR (95 % CI)**	**AOR (95 % CI)**	***P*-value**
		**Yes (** ***n*** **/%)**	**No (** ***n*** **/%)**			
Sex	Male	27 (4.5)	571 (95.5)	1	1	
	Female	17 (3.0)	549 (97.0)	0.65 (0.35–1.21)	0.52 (0.21–1.27)	0.150
Age group	< 45 years	17 (1.8)	924 (98.2)	1	1	
	≥45 years	27 (12.1)	196 (87.9)	7.49 (4.00–14.00)	3.97 (1.80–8.74)	0.001
Level of education	≤ Secondary school	27 (6.4)	398 (93.7)	1	1	
	College and above	17 (2.3)	722 (97.7)	0.35 (0.19–0.65)	0.39 (0.17–0.89)	0.025
Marital status	Never married	5 (1.2)	422 (98.8)	1	1	
	Married	35 (5.3)	632 (94.7)	4.67 (1.82–12.03)	1.44 (0.46–4.53)	0.553
	Divorced/widowed	4 (5.7)	66 (94.3)	5.12 (1.34–19.54)	1.67 (0.30–9.42)	0.560
Ever cigarette smoking	Non-smoker	34 (3.3)	999 (96.7)	1	1	
	Smoker	10 (7.6)	121 (92.4)	2.69 (1.35–5.35)	0.97 (0.39–2.43)	0.950
Serving fruits and vegetables per day	None	20 (29.0)	49 (71.0)	1	1	
	At least once	24 (2.2)	1,071 (97.8)	0.06 (0.03–0.11)	0.10 (0.04–0.22)	0.000
Khat chewing	No	16(2.1)	752 (97.9)	1	1	
	Yes	28(7.2)	368 (92.8)	3.18 (1.73–5.83)	2.76 (1.23–6.18)	0.014
Body Mass Index	< 25 kg/m2	8 (1.1)	743 (98.9)	1	1	
	≥ 25 kg/m2	36 (8.7)	377 (91.3)	8.87 (4.08–19.27)	5.11 (2.06–12.66)	0.000
Sedentary behavior	< 8 h per day	12 (1.3)	916 (98.7)	1	1	
	≥ 8 h per day	32 (13.6)	204 (86.4)	11.97 (6.06–23.65)	6.44 (2.89–14.34)	0.000
Alcohol consumption	No	16 (2.7)	587 (97.3)	1	1	
	Yes	28 (5.0)	533 (95.0)	1.93 (0.03–3.60)	1.30 (0.58–2.88)	0.521
Family history of HPN	No	31 (3.3)	912 (96.7)	1	1	
	Yes	13(5.9)	208 (94.1)	1.84 (0.95–3.58)	1.47 (0.58–3.70)	0.418
Family history of DM	No	35 (3.3)	1,040 (96.7)	1	1	
	Yes	9 (10.1)	80 (89.9)	3.34 (1.55–7.20)	1.47 (0.49–4.44)	0.494

## Discussion

This study found the overall prevalence of HTN, T2DM, and co-occurrence of HTN and T2DM were 27, 8, and 4%, respectively. Co-occurrence of HTN and T2DM was significantly increased with advanced ages, body mass index >25 kg/m^2^, khat chewers, those with sedentary behaviors, and a higher level of education, while co-occurrence of HTN and T2DM was significantly lower among individuals those who reported consuming one or more servings of fruits and vegetables per day.

In this study, more than one in four employees had HTN. This finding is consistent with a study conducted in Wollo, North Ethiopia (34); Sidama, South Ethiopia (35); and Debre Birhan, North Ethiopia (36); and Addis Ababa, Ethiopia (25). However, the finding of this study is higher than that of the study conducted in Wolaita, Southern Ethiopia (37); Hawassa, Southern Ethiopia (38); and Togo (39). This may be due to the difference in study participant characteristics and study setting. For instance, our study population is university employees, whereas the others included working adults. In addition, the high prevalence of hypertension in our study could be due to a more sedentary lifestyle, increased pressure from the high workload, and changes in dietary patterns (40). However, the finding of this study is lower than study in Southern (41), northwestern (42) and northern Ethiopia (43), southern Nigeria (44) and Bangladeshi (45). This difference may be due to the sample size and setting of the study. Changing nutritional intake combined with increasingly sedentary lifestyles was reported to increase the emergence of chronic diseases such as hypertension in developing countries (9, 10).

Similarly, ~8% of the study participants were found to have T2DM. This is consistent with the study conducted in northern Ethiopia (43) and Addis Ababa (46), but the percentage of prevalence was higher than the study conducted in southern Nigeria (44) and Taiwan (47). This may be due to a high level of physical inactivity and poor consumption of fruits and vegetables in the study area and an unhealthy diet due to the entry site of a processed and packed diet. However, this finding is lower than the percentage of prevalence found in eastern Sudan (48) and north Sudan (49), Spain (50), and France (51). This may be due to working types and conditions, income, and educational status in urban centers of high-income countries that predispose them to stay home, consume unhealthy diets, and have multiple behavioral risk factors.

This study showed that ~4% of the study participants had both HTN and T2DM. This finding is consistent with studies conducted in Bangladesh (19) and China (22). This finding coincides with the fact that the lifestyle of working adults in Ethiopia has radically changed in the last decade due to the changing working environment, concentration of the middle-aged population, urban dwellers, risk of sedentarism, less physical activities in the workplace, better access to technology, and leisure lifestyle status. However, this finding is lower than the percentage of prevalence found in Ghana (52) and the USA (53). This difference may be due to the study units used (public employees in this study and any adult in the other two studies) and other socio-demographic and dietary patterns of the study population. Accumulating evidence has shown that hypertension affects the majority of individuals with diabetes mellitus which dramatically increases the risk of morbidity and mortality from cardiovascular disease (54, 55). The co-existence of HTN and T2DM is a major contributor to the development and progression of microvascular and macrovascular complications, which in turn complicates the treatment strategy and increases healthcare costs.

This study showed that advanced age was significantly associated with the co-occurrence of HTN and T2DM. This finding is consistent with studies conducted in North Wollo Zone, Amhara Region (34); Wolaita Zone, Southern Ethiopia (37); Northwest Ethiopia (42) and Southern Ethiopia (38); Sidama Zone, South Ethiopia (35); and Ghana (56) where older age or advance in age is found to increase the risk of hypertension and/or diabetes. Over-abundant food consumption combined with a more sedentary lifestyle among older people mostly accounts for the increase in chronic non-communicable diseases (57, 58).

This study demonstrates that the co-occurrence of HTN and T2DM was 61% lower among public servants with a college and above education level. Consistent with this finding, studies showed that people who have a low socioeconomic status have a higher risk of non-communicable diseases (NCDs) than more advantaged groups. Social inequalities accounted for more than half of inequalities in major NCDs (59–61). Being educated is more effective in reducing unhealthy diet, substance use, physical inactivity, and better access to healthcare for timely diagnosis and treatment (62).

Study participants who chew khat were approximately three times more likely to have the co-occurrence of HTN and T2DM as compared to non-khat chewers. This finding is consistent with a study conducted in Southern Ethiopia (35) and Southwest Ethiopia (63), which indicates that the risk of either hypertension or diabetes was increased among people who chewed khat. Evidence shows that khat chewing has a significant effect on increasing fasting plasma glucose (64) and mean blood pressure (65).

Similarly, our study showed that employees who had sedentary behavior (≥8 h per day) are more likely to have the co-occurrence of HTN and T2DM than their counterparts. This finding is consistent with studies conducted in Southern Ethiopia (41) and China (66). Insufficient physical activity is one important cause of most chronic diseases and results in substantial disease in quality of life (67, 68). Physical activity primarily prevents or delays chronic diseases. Preventing sedentary behavior with increased promotion of physical activity needs to be part of any healthcare system. BMI≥25 Kg/m^2^ was among the modifiable risk factors found to increase co-occurrence of HTN and T2DM in this study. Similar studies in Ethiopia showed BMI≥25 Kg/m^2^ or being overweight or obese (35, 36, 38, 69) increased the risk of HTN or diabetes or both. This finding is also consistent with a study by Tseng where “BMI/obesity is significantly linked to blood pressure/hypertension throughout the range of BMI in diabetic patients in either sex regardless of a previous hypertension history” (70). Dietary mediators seem to play a significant role in the pathogenesis of cardiovascular disease, among different socioeconomic layers (71).

In this study, study participants who had servings of fruits and vegetables at least once per day were less likely to have both HTN and T2DM as compared to their counterparts who do not consume servings of fruits and vegetables. A study conducted in Ghana (56) showed high fruit intake was associated with a lower risk of hypertension. Several studies showed that unhealthy diets and physical inactivity are well-recognized modifiable behavioral risk factors for NCDs (72–74). Studies showed that the majority of cardiovascular diseases can be attributed to major risk factors, such as low intake of fruits and vegetables rather than consuming more foods that are nutritionally poor and consuming energy-dense foods high in sugar and/or saturated fats or excessively salty (22). People with type 2 diabetes are encouraged to optimize dietary patterns to reduce their risk for cardiovascular diseases, such as diabetes and related comorbidities (75). This study revealed very critical evidence on the co-occurrence of HTN and T2DM, which can be very useful for policymakers in low- and middle-income countries for early interventions to limit the pace of such non-communicable diseases.

## Limitations and strengths of the study

The limitation of this study is reliance on self-reporting, which contributes to recall and social desirability bias that potentially underestimates behavioral factors. This study may not be generalizable to all employees in Eastern Ethiopia as the study population was drawn only from one institution. However, the strengths of this study include large sample size and a standardized questionnaire based on World Health Organization's (WHO) STEP approach to collect data. Moreover, this study was the first of its kind among university employees in Ethiopia and can be fairly generalized for this category of workers in areas where there are contextual working adults.

## Conclusion

Approximately 4% of Haramaya University employees had co-occurrence of HTN and T2DM. Factors associated with the co-occurrence of HTN and T2DM were older age, overweight/obesity, sedentary behavior, higher level of education, consumption of khat, and less intake of fruits and vegetables. These findings call for the health promotion interventional strategies targeting the aforementioned determinants. Increasing awareness of controlled consumption of “khat,” lifestyle modifications, and strengthening job periodic screening programs of high-risk populations are recommended.

## Data availability statement

The original contributions presented in the study are included in the article/supplementary material, further inquiries can be directed to the corresponding author.

## Ethics statement

The studies involving human participants were reviewed and approved by Institutional Health Research Ethics Review Committee (Ref. No. IHRERC/196/2018) of Haramaya University, College of Health and Medical Sciences. The patients/participants provided their written informed consent to participate in this study.

## Author contributions

AM conceived the study and drafted the proposal. AT, AM, and LR had substantial contributions to the study design and development of the data collection checklist and drafted, wrote, and corrected the manuscript. All authors read the manuscript and approved it.
